# Computational Evolutionary Analysis of the Overlapped *Surface* (S) and *Polymerase* (P) Region in Hepatitis B Virus Indicates the Spacer Domain in P Is Crucial for Survival

**DOI:** 10.1371/journal.pone.0060098

**Published:** 2013-04-05

**Authors:** Ping Chen, Yun Gan, Na Han, Wei Fang, Jiafu Li, Fei Zhao, Kanghong Hu, Simon Rayner

**Affiliations:** 1 Key Laboratory of Agricultural and Environmental Microbiology, Wuhan Institute of Virology, Chinese Academy of Sciences, Wuhan, China; 2 State Key Laboratory of Virology, Wuhan Institute of Virology, Chinese Academy of Sciences, Wuhan, China; 3 Department of Obstetrics and Gynecology, Zhongnan Hospital of Wuhan University, Wuhan, China; 4 Biomedical Center, Hubei University of Technology, Wuhan, China; Centers for Disease Control and Prevention, United States of America

## Abstract

**Introduction:**

The Hepatitis B Virus (HBV) genome contains four ORFs, S (surface), P (polymerase), C (core) and X. S is completely overlapped by P and as a consequence the overlapping region is subject to distinctive evolutionary constraints compared to the remainder of the genome. Specifically, a non-synonymous substitution in one coding frame may produce a synonymous substitution in the alternative frame, suggesting a possible conflict between requirements for diversifying and purifying forces. To examine how these contrasting requirements are balanced within this region, we investigated the relationship amongst positive selection sites, conserved regions, epitopes and elements of protein structure to consider how HBV balances the contrasting evolutionary pressures.

**Methodology/Results:**

323 HBV genotype D genome sequences were collected and analyzed to identify sites under positive selection and highly conserved regions. Epitopes sequences were retrieved from previously published experimental studies stored in the Immune Epitope Database. Predicted secondary structures were used to investigate the association between structure and conservation. Entropy was used as a measure of conservation and bivariate logistic regression was used to investigate the relationship between positive selection/conserved sites and epitope/secondary structure regions. Our results indicate: (i) conservation in S is primarily dictated by α-helix elements in the protein structure, (ii) variable residues are mainly located in PreS, the major hydrophilic region (MHR) and the C-terminus, (iii) epitopes in S, which are directly targeted by the host immune system, are significantly associated with sites under positive selection.

**Conclusions:**

The highly variable spacer domain in P, which corresponds to PreS in S, appears to act as a harbor for the accumulation of mutations that can provide flexibility for conformational changes and responding to immune pressure.

## Introduction

Both hepatitis B virus (HBV) and hepatitis C virus (HCV) cause persistent liver infection, but the two viruses are notably different in terms of replication strategy and host interaction, as well as their global impact on public health [1], [2]. 170 million people are estimated to be infected worldwide with HCV, with 70–90% of infected individuals becoming chronically infected [3]. In contrast, more than 350 million people are estimated to be globally infected with HBV but more than 95% of cases will result in viral clearance [4]. The viruses also possess strikingly different genome arrangements. HCV, a member of the flaviviridae family, possesses a positive strand RNA genome of ∼9.6 Kb encoding a polyprotein that is co- and post-translationally processed to form three structural (core, envelope 1 (E1) and envelope 2 (E2/p7)) and six non-structural proteins (NS2, NS3, NS4A, NS4B, NS5A & NS5B) [5]. HBV, on the other hand, is the smallest known DNA virus with a genome only 3.2 kb in length. The genome comprises four open reading frames (ORF): core (C), polymerase (P), surface (S) and X. All four ORFs are overlapped completely or partially. Specifically, S is encompassed entirely by P. Gene overlapping is a common strategy adopted by many viruses to reduce their genome size and maximize their encoding capacity. However, this inevitably constrains the independent evolution of the individual reading frames as a mutation with little effect on one gene may cause severe or even fatal changes on the cognate overlapping gene. Thus, in an overlapping region, if one gene undergoes adaptive evolution (positive selection) with a high ratio of non-synonymous nucleotide mutations (*d_n_/d_s_* >1), the cognate gene often undergoes purifying selection (negative selection; *d_n_/d_s_* <1). This has been observed in many different viruses such as simian immunodeficiency virus [6], potato leaf roll virus [7] and human papilloma virus [8].

Studies on the variation in HCV sequences indicate that E1 & E2 (which interact with the host immune system) possess greater variation compared to the NS5B protein, which encodes the RNA-dependent RNA polymerase, and this disparity may reflect the differing functional roles of these two proteins [9]–[11]. In HBV, however, S and P shared an overlapping segment of DNA sequence, raising the question of whether these proteins face similar competing requirements and, if so, how they resolve this apparent conflict. From a structural perspective, the HBV P protein comprises a terminal protein (TP) domain, a reverse transcriptase (RT) domain, an RNase H (RH) domain and a spacer domain [12]. Of these structures, the TP, RT and RH domains are conserved, while the spacer domain is highly variable (for review see [13], [14]). Previous genetic studies have suggested that the spacer, which acts a tether between TP and RT, is dispensable as it has little effect on replication competence [12], [13]. The S ORF encodes three surface proteins termed large (L), middle (M), and small (S) protein. The M protein is comprised of the S and PreS2 domains. The L protein includes another N-terminus genotype-dependent domain termed PreS1. PreS1 is essential for viral entry and infection. In particular, amino acids 2–48 act as the recognition site for a hepatocyte-specific receptor [15]. The S protein is predicted to function as a membrane spanning protein and contains four trans-membrane (TM) regions, each consisting of an α-helix structure [16]. Compared with the variable PreS domain (including PreS1 and PreS2), the TM regions, which maintains the stability of protein structure, are conserved [16]. However, the hydrophilic loops between the α-helices harbor more variable amino acid residues. Moreover, variation in epitopes may aid virus escape from the host immune system. The T cell and B cell epitopes in the S protein (HBsAg) targeted by the host immune system are mainly concentrated in these loop regions, including the major hydrophilic region (MHR, amino acids 99–160) which contains a conformational B cell epitope cluster. In addition, the core of MHR contains the “a” determinant (residues 121–147) which is the region primarily associated with induction of a protective humoral immune response [17], [18].

The selection pressure exerted by the host immune system can be focused on the epitope regions, although this pressure varies over the course of an HBV infection [19], [20]. Specifically, the humoral immune (B cell mediated) response to the S protein plays a relevant role in the clearance of infectious HBV particles, whereas cellular immune (T cell mediated) responses contribute to the elimination of infected hepatocytes [19], [21]. The studies on immunopathogenesis of S are extensive, and hence there are large quantities of associated epitope data. In contrast, as a consequence of the fewer reports on both the humoral immune response and the cytotoxic T cell (CTL) response, there are relatively less data available on epitopes data in P [22]–[27]. Moreover, the higher levels of conservation in P may restrict viral escape via mutations in epitopes [24]. Thus, the correlation between epitope of P protein and selection pressure is not considered in this report.

Several studies have investigated the effects of these functional constraints in HBV [28]–[30]. Although sequence evolution in the overlapped P and S regions is constrained [28], Zaaijer *et al*. (2007) [29] proposed that HBV is able to use the degeneracy in the genetic code to overcome these restraints. Due to the frame shift between the coding regions for P and S, the first position in the P codon corresponds to the third position in the S codon (P_1_S_3_), the second position in the P codon corresponds to the first position in the S codon (P_2_S_1_), and the third position in the P codon correspond to the second position in the S codon (P_3_S_2_). Thus, a synonymous mutation in P is able to produce a corresponding non-synonymous mutation in S (P_3_S_2_), which can produce an amino acid change in S but conserve the corresponding site in P, satisfying the constraints on both genes. Furthermore, the study found that the most of changes take place in P_1_S_3_ (P) or P_3_S_2_ (S) and the nucleotide mutations in P_2_S_1_ are rare. In a more recent study, negative selection was simultaneously detected in both the overlapped P and S genes [30]. However, the dataset was composed of a single study set of 33 patients and hence involved analysis of a relatively small number of sequences.

In this study, we reinvestigated the overlapping P and S regions using a dataset, significantly larger compared to previous studies and considered how the observed variation in this region was related to what is known about the respective functions of the two proteins[28]–[30]. We examined the correlation between conservation and structured protein domains, as well as the relationship between positive selection sites and epitope regions and consider how these competing requirements help to shape the HBV genome and impact the life cycle of the virus.

## Materials and Methods

### Data Collection and Preparation

Up to May 2012, all available full-length human HBV genome sequences (3165) were retrieved from GenBank. All sequences were genotyped using the software tool HBV STAR (http://www.vgb.ucl.ac.uk/starn.shtml) [31]. To rule out the possibility of inter-genotype recombination, only sequences that had been previously established to be non-recombinant were used as references. All inter-genotype recombinant sequences, sequences with ambiguous characters (non-standard nucleotides or amino acids) were removed. This left a final dataset containing 2236 genotyped human HBV genome sequences (genotype A, n = 300; genotype B, n = 604; genotype C, n = 717; genotype D, n = 323; genotype E, n = 181; genotype F, n = 59; genotype G, n = 25; genotype H, n = 27). Finally, the associated publications for the 323 genotype D sequences were checked to ensure they were from drug naive subjects who did not test positive for coinfection with other viruses such as HIV or HCV. These 323 sequences were used in the subsequent analysis. The known epitopes of HBV surface antigens (L, M, S), including T cell epitopes and B cell (antibody) epitopes, were retrieved from the immune epitope database (IEDB; http://www.immuneepitope.org/). Background information (i.e., accession numbers and genotypes), as well as the epitope information (epitope ID and linear sequence), are presented in Table S1 and Table S2 respectively. The overlapped P and S sequences, comprising amino acid and DNA sequences, were extracted from HBV whole genome sequences, and aligned using ClustalW v2.0 (http://www.clustal.org/download/) [32]. The alignments of DNA sequences were manually adjusted according to the amino acids alignment by using MEGA version 5.0 (http://www.megasoftware.net/) [33] and sequences with large alignment gaps were removed. Both the polymerase and large surface protein of genotype D have a deletion (11 amino acids in length) compared with the remaining genotypes (A, B, C, E-H). This indel is highly conserved and it has little effect on the results of the following analyses, therefore it was treated as an alignment gap and removed. For consistency, the large surface protein and the corresponding P codons within the overlapping region were numbered from 1 to 389 according to the genotype D reference sequence reported in a previous study by Myers *et al.* [31] (accession no. X65259).

### Investigation of Positive Selection Pressure

Two different tools were used to investigate the positive selection pressure. First of all, six different codon-based substitution models (M0 (one ratio (ω)), M1 (neutral), M2 (positive selection), M3 (discrete), M7 (beta) and M8 (beta & ω >1)), implemented in the *Codeml* program in the PAML software package version 4.0 (http://abacus.gene.ucl.ac.uk/software/paml.html) [34], were used to test the ratio of non-synonymous to synonymous nucleotide substitutions (*d_n_*/*d_s_*). The likelihood ratio test (LRT) and Bayes Empirical Bayes (BEB) [35] statistical tests applied by PAML were used to determine the most suitable models, following the method described in the user manual. The multi-partition fixed effects likelihood (FEL) method implemented in the Hyphy [36] software package on the online server (http://www.datamonkey.org/) was then used to predict positive selection sites.

### Identification of Conserved and Highly Variable Regions

The entropy values (H_0_) [37] varying from 0 (100% conserved) to 1 (all 20 amino acids present at equal frequency), as well as the frequency of conserved residues, were used to quantify the variation of amino acids in the overlapped P and S proteins. The Shannon entropy (*H_0_*) of each site was calculated according to the following equation
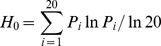
where *P_i_* is the probability of amino acid *i* occurring at the site [37].

### Protein Structure Modeling

The 3D structures of the P and S proteins were predicted based on homology modeling or *de novo* modeling. Both the consensus sequences of P and S were derived from the alignments of genotype D. Using the HIV RT structure 1T05 (2.8 Å) as a template, an initial model of HBV RT was generated using Modeller (http://salilab.org/modeller/) [38]. Once generated, the model was transferred to Chimera (http://www.cgl.ucsf.edu/chimera/download.html) [39] for model refinement based on energy minimization with an Amber99 force field. Model quality was evaluated using PROCHECK. Due to lack of a suitable template, the 3D model of the S protein was predicted using the online server I-TASSER based on an algorithm consisting of consecutive steps of threading and fragment assembly to obtain an estimated structure with the lowest energy [40]. According to the predicted tertiary structure, the secondary structure of the P protein was generated using the DSSP software package (http://swift.cmbi.ru.nl/gv/dssp/) [41], while the secondary structure of S was predicted using the Raptox-SS8 software package (http://ttic.uchicago.edu/~zywang/RaptorX-SS8/) [42]. In the following analyses, amino acids located in an α-helix or β-sheet were considered part of the structured region and the remaining residues located in loops, coils and turns were associated with unstructured regions.

### Statistical Testing

The associations between positive selection sites and epitopes, and between conservation and structured protein domains (α-helix and β-sheet), were evaluated using the Fisher’s exact test based on a series of contingency tables. The data was further investigated using bivariate logistic regression to investigate the relationship between positive selection/conserved sites and epitope/secondary structure regions. All these analyses were performed in R, version 2.15.0 (http://www.r-project.org/). Full details are provided in Table S4.

## Results

### Identification of Positive Selection in P and S

The six site models implemented in the PAML software package were compared (M3 to M0, M2 to M1, and M8 to M7) by the likelihood ratio test. For both proteins the M3 (discrete), M2 (positive), and M8 (β and ω >1) models were selected (P<0.01) (Table S3), suggesting varying selection pressure occurs at individual sites throughout the HBV P and S overlapping region. Using HyPhy [36], sites under positive selection were detected in both the P and S proteins. In genotype D, 27 sites, corresponding to 3.2% of the 832 amino acids of the full length P protein, were found to be under positive selection; for the P and S overlapped region 3.9% of the sites were predicted to be under positive selection (15 sites out of 389). In the S protein, the proportion was 5.1% (20 residues). Moreover, in the S and P overlapped region, the sites predicted to be under positive selection are not randomly distributed throughout the defined domains. For P, 86.7 percent (13 out of 15) of the positive selection sites are located in the spacer domain, while the RT domain only contains two sites (13.3%). Similarly, for S, 85% (17 out of 20) of the positive selection sites are concentrated in PreS (6), the major hydrophilic region (MHR) (6) and the C-terminus (5), and the remaining regions only contained 3 sites.

**Figure 1:**
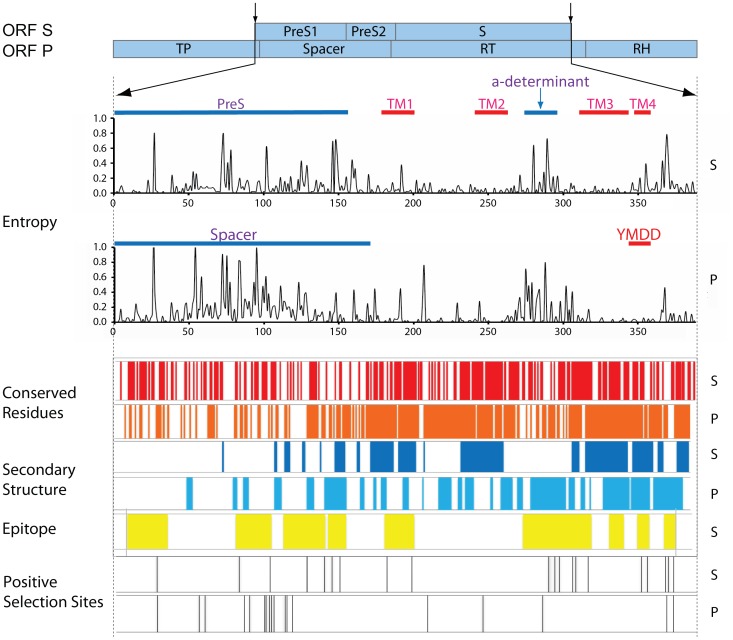
Map of the Overlapping Region of the S and P Genes. The line at the top shows a schematic of the major components of the S and P genes. The arrows above mark the location of the overlapping regions of the two genes. The spacer domain in P more or less corresponds to the PreS (PreS1+ PreS2) domain in S, whereas the RT domain in P corresponds to the S domain in S. The plots below show the variation within the overlapping region and the location of specific features for both genes. First row: Entropy plots for S (upper plot) and P (lower plot). The X-axis denotes the codon position (1–389) and refers to the position within the overlapping region. The Y-axis denotes the entropy of the sites, with a higher value representing a more variable codon. The location of important regions within each gene is marked above each plot. For S these are PreS, a-determinant and four transmembrane regions TM1 to TM4. For P these are Spacer and the YMDD motif. In S, the variable residues are mainly located in PreS, the “a” determinant and at the C-terminus, while the trans-membrane regions are relatively well conserved. In P, the Spacer domain and the region corresponding to “a” determinant are highly variable, while the most conserved codons are locate within and near the YMDD motif. Row 2 shows the location of highly conserved codons for S (upper plot) and P (lower plot), based on the entropy plots. Row 3 shows the location of predicted secondary structure features (alpha helix and beta sheets) based on predicted protein structures for S (upper plot) and P (lower plot). Row 4 shows the location of epitopes within the S protein. Row 5 shows the sites predicted to be under positive selection for S (upper plot) and P (lower plot).

To investigate whether the selection pressure acts predominantly on viral epitopes, known T cell and B cell (antibody) epitopes, together with the sites under positive selection, were mapped on to the protein sequence for the S gene (Figure 1). In S, there was a significant association (p = 0.01) (Table 1B) between epitopes and sites under positive selection in the S protein.

**Table 1:** Association between conservation, secondary structure, positive selection sites and epitopes performed by Fisher’s exact test.

	OR	p-value
(A) P protein (Conservation VS. 2^nd^ structure)		
α-helix	2.83	3.01e-05
β-sheet	1.94	0.09
(B) S protein (Positive selection VS. epitopes)		
	Infinite	0.01
(C) S protein (Variation VS. epitopes)		
	0.47	0.02
(D) S protein (Conservation VS 2^nd^ structure)		
α-helix	1.96	0.01
β-sheet	1.06	NS

OR: odd ratio; NS: not significant.

Association between (A) Conserved sites and secondary structure (B) sites under positive selection and epitopes and (C) variation (entropy) and epitopes for S protein. (D) association between Conserved sites and secondary structure for P protein. The odds ratio provides a measure of the association between two specified variables. For example, in (A) conserved sites have a strong association with α-helices both in the S (OR = 1.96, P = 0.01) and the P protein (OR = 2.83, P = 3.01e-05<0.01), a weak association with β-sheets in the P protein (OR = 1.94, P = 0.09), but have no significant association between conserved sites and β-sheets in the S protein, indicating the α-helices are highly conserved and the β-sheets can accommodate more variable residues.

### Prediction of Secondary and Tertiary Structures of the P and S Proteins

**Figure 2:**
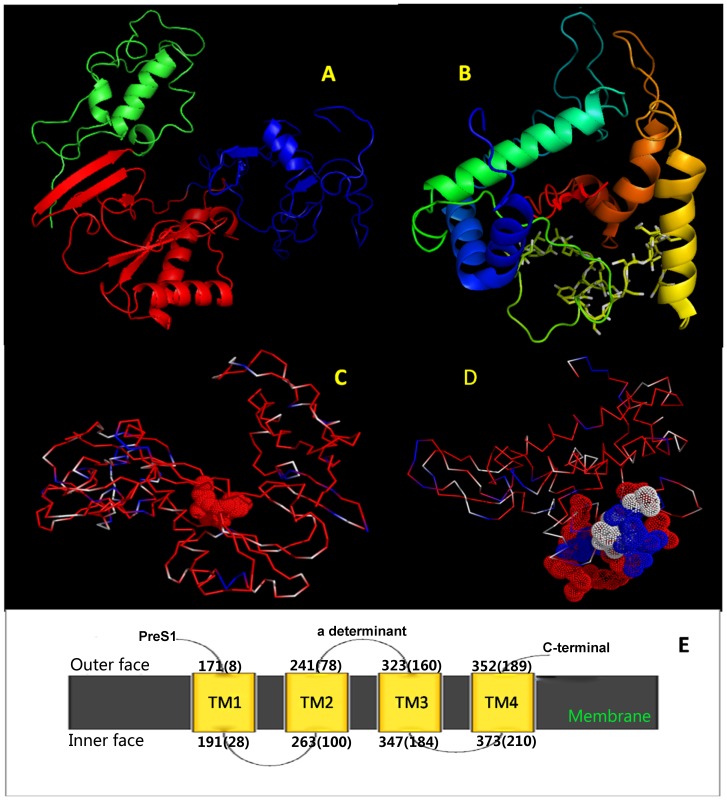
Predicted 3D Structures for the S and P Proteins. A) The predicted 3D model of the HBV RT based on the HIV RT structure which folds in the classic “right hand” shape with fingers (blue), palm (red) and thumb (green) subdomains. The finger and thumb subdomains are primarily composed of α-helices, whereas the palm regions mainly comprises α-helix and β-sheets. B) The predicted 3D model for S. S contains four long α-helices which constitute the trans-membrane (TM) regions. These are colored blue (TM1), green (TM2), yellow (TM3) and brown (TM4) respectively. These α-helices are each separated by loops and the “a” determinant located in the loop between TM2 and TM3. C) The spatial distribution of conserved and variable residues in HBV RT. The highly conserved residues are colored red, the highly variable residues are colored blue, and the remaining residues are colored white. The majority of residues are conserved. Furthermore, the most conserved residues are clustered within and near the YMDD motif (marked as red spheres). D) The spatial distribution of conserved and variable residues in S. Red and blue indicate the most highly conserved and most variable residues respectively, the remaining residues are colored white. The “a” determinant (marked with spheres with the same colour scheme to show variability) harbors many B cell epitopes and contains many highly variable sites (blue spheres). Compared to P, the distribution of variable sites in S appears to be more diffuse. E) Schematic of secondary structure of S. S has four membrane spanning regions (TM1–TM4). The N-terminus, C-terminus and “a” determinant are located on the outer face of the membrane. Coordinates of the membrane spanning regions are shown for inner and outer face. Top coordinate corresponds to the position within the pre-S1, coordinates in parentheses correspond to the position within the small S.

In order to investigate the associations between protein structure and positive selection, the individual secondary and tertiary structures of S and P were modeled. Since the structures of P and S have not been experimentally determined, we generated 3D models for both proteins based on the consensus sequences derived from genotype D. The full alignment of the HBV RT domain with the corresponding HIV-1 RT was consistent with previously published estimates of the HBV RT structure [43]. Based on this alignment, a final model was generated (Figure 2A). Consistent with the solved structure for the HIV-1 RT and other DNA-dependent DNA polymerase (DDDP) or RNA-dependent DNA polymerase (RDDP), the HBV RT model folds in a classic “right hand” shape with fingers, palm and thumb subdomains. The finger (rt1-79 and 145–195) and thumb (290–376) subdomains are mainly composed of α-helices, while the palm (80–116 and 196–289) subdomains which constitute the catalytic “core” of polymerase are dominated by β-sheets and α-helices. The palm contains the YMDD motif, which is associated with mutations after long-term antiviral treatment with nucleoside analogues (NA) such as Lamivudine, Entecavir. The *de novo* prediction for S contains four long α-helices which are considered to constitute the trans-membrane (TM) regions (Figure 2B, colored blue, green, yellow and red respectively). A schematic of S based on secondary structure and a previous published study [16] is shown in Figure 2E. The four membrane spanning regions are termed as TM1 (8–28), TM2 (78–100), TM3 (160–184) and TM4 (189–210) respectively and a major loop between TM2 and TM3 in the extracellular spacer harbors the “a” determinant.

### Pattern of Conserved and Variable Amino Acids

The entropy and frequency of consensus amino acids were used to estimate the degree of variability within each protein. A plot of entropy superimposed over the sequence shows that the majority of variable residues in S are mainly concentrated in PreS, the “a” determinant and the C-terminus (Figure 1). In contrast, most of the variable residues in P are located within the spacer region and codons 290–310 which correspond to the “a” determinant in S (Figure 1, Figure 2D). If we define a site to be conserved when more than 95% of the sequences harbor the same amino acid, we find that 73.3% of the amino acid residues for the P protein are conserved, whereas for S, 71.7% of the sites are conserved. If we set the threshold value to 99%, the percent of conserved residues are 62% (P) versus 55% (S). When we mapped the conserved residues to the predicted protein structure, at a 95% consensus cut off, 77% of the residues located in the structured regions of P were conserved (α-helix (78% of residues conserved) and β-sheet (71% of residues conserved)). The result of the Fisher’s exact test (Table 1) indicated a significant restriction in sequence variability in the α-helix domains (odd ratio (OR) = 2.83, P = 3.01e^−5^) but a relaxed restriction within the β-sheet domains (OR = 1.94, P = 0.09) (Table 1A). For the S protein, the α-helix exhibited a strong association with conservation of virus sequence (OR = 1.96, P = 0.01), but there was no such association identified for β-sheets (Table 1D). When the variable and conserved residues are mapped on the 3D structures for P and S, they show different spatial distributions. In P, the highly conserved residues cluster in the catalytic core near the YMDD motif (Figure 2C); in contrast, the variable residues in S are mainly located in the loops between the long α-helices which are thought to constitute the trans-membrane regions (Figure 2D).

### Correlations among Protein Structure, Epitopes, and Amino Acid Variability

Constraining and diversifying forces are in conflict and each contributes to shaping a viral genome. We therefore used bivariate logistic regression to investigate the magnitude of contributions from both events (i.e., conservation and positive selection) (Table 2). The results indicate that constraints occur primarily in domains located in α-helices (OR = 1.89, p = 0.04), but no significant association between conserved regions and epitopes (p>0.05) is demonstrated (Table 2A). However, the Fisher’s exact test indicates significant correlation between variable sites and T cell and antibody epitopes (OR = 0.47, p = 0.02) (Table 1C), implying these regions tolerate more variability. For the positive site bivariate regression analysis the regression model is rejected (Table 2B), but there is significantly less positive selection occurring in α-helix domains (Figure 1). This is consistent with the increased conservation observed in these regions. Also, we found an increased number of residues under positive selection in T cell and antibody epitopes which is consistent with the significantly increased variability in these domains (Figure 1).

**Table 2:** Bivariate logistic regression analysis for association with (A) conservation, or (B) positive selection sites in S protein.

	Coef.	Std. Err.	OR	p-value	95% CI
(A) Conservation (P(> χ^2^ ) = 0.008)					
X1 (α-helix+β-sheet)	0.64	0.59	1.89	0.041	1.03–3.47
X2 (epitopes)	0.49	0.43	1.64	0.058	0.98–2.72
(B) Positive selection (P(> χ^2^) >0.05)					
X1 (α-helix+β-sheet)	0.14	0.38	na	0.71^NS^	na
X2 (epitopes)	16.24	887.44	na	0.98^NS^	na

Coef: coefficient; Std. Err.: standard error; OR: odd ratio; CI: confidence interval; NS: not significant; na: not applicable. Logistic regression analysis was carried out with conservation as the predicted variable. (A) The estimated coefficients suggest that the conservation is significantly associated with structural region (α-helix+β-sheet) (Coef. = 0.64 with p = 0.041<0.5), but has no significant association with epitopes (Coef. = 0.49 with p = 0.058>0.05). This result is consistent with the results of Fisher’s exact test. (B) The logistic model with X1 and X2 as predictor variables is not significant due to the fact P(> χ^2^) >0.05.

## Discussion

Gene overlapping is a common occurrence in viruses. In this way, a virus can minimize its genomic size, effecting a more economical replication cycle. The trade-off is that within an overlapping region, nucleotide substitutions may result in simultaneous amino acid mutations in the two distinct proteins encoded by the same nucleotide sequence. Consequently, this restricts the independent evolution of overlapped genes. How natural selection acts on the different viral proteins within an overlapping region continues to interest evolutionary biologists and virologists. Adaptive (positive) selection in one protein contrasted by purifying (negative) selection in the other overlapping protein has been observed in several viruses including simian immunodeficiency virus, potato leaf roll virus and human papilloma virus [6]–[8].

HBV is the infectious pathogen responsible for hepatitis B and possesses a highly compact DNA virus with half of its genome overlapped. The first identification of distinctive evolutionary constraints acting on the HBV genome was reported in 1997 by Mizokami *et al*. who analyzed 27 HBV strains [28]. They found a lower rate of nonsynonymous substitutions to synonymous substitutions (*d_n_/d_s_*) in the non-overlapped region compared to the overlapped region. Subsequently, Zaaijer *et al.* showed the overlapping polymerase and surface protein were undergoing adaptive selection but proposed they were nevertheless, to a certain degree, evolving independently [29]. Most recently, based on a different dataset, van der Klundert *et al.* detected negative selection acting on the HBV genome [30]. In this study, we reexamined the adaptive evolution acting in the overlapping P and S regions using a significantly larger dataset compared to earlier studies. In addition to investigating *d_n_/d_s_* we also examined the association between conserved sites and structured protein domains, and between sites under positive selection and epitope regions. Although the crystal structure of both proteins remains to be determined, in this study we primarily focused on the secondary structure which can be predicted with higher confidence (∼80%) [44]–[46]. Furthermore, our structure prediction for P is based on the tertiary RT structure of the HIV RT, which contains many regions that are well conserved between the two viruses, further increasing the accuracy of our prediction.

As a DNA-dependent DNA polymerase (DDDP) and an RNA-dependent DNA polymerase (RDDP), the HBV polymerase requires sequence conservation to implement its normal catalytic function. In particular, a highly conserved catalytic core in the proximity of the YMDD motif is crucial for replication competence, even a single amino acid mutation may severely decrease the catalytic activity of polymerase. Long-term antiviral treatment with nucleoside analogues (NA) such as Lamivudine and Entecavir often results in YMDD mutations and mutations have occasionally been reported in Tenofovir treatment [47], [48]. Although they can tolerate antivirals, the mutants may reduce their replication competence in comparison to the wild type virus [49]. To remove any bias that might result from anthropogenic selection so as to focus on investigating the evolutionary pattern under natural selection pressure, sequences with antiviral therapy were excluded.

In the HBV P and S overlapping region, several interrelated factors including structural, immunological and evolutionary constraints affect relative gene domain arrangement and genetic variation. The requirement for maintaining structural protein elements (α-helix and β-sheet) appears to govern the conservation of residues and restrict the virus variation within the genome. In particular, as a stable and important structural element, few nonsynonymous substitutions exist in the α-helices. In contrast, the β-sheets can accommodate more variation which is consistent with results from an analysis of structural restraints in HIV [50].

Many sites under positive selection were detected in both genes in this study, indicating they are both undergoing adaptive evolution and suggesting that they each play important roles in HBV survival. The adaptive evolution identified in S was almost exclusively located within the epitope regions, suggesting a role associated with evasion of host immune system. However, we identified additional sites outside the epitopes with weaker statistical support, that may also be associated with alternative roles within the virus life cycle and which would warrant further investigation. [51], [52]. On the other hand, the positive selection sites in P were mainly located in the spacer domain which corresponds to PreS in S, suggesting that this “dispensable” spacer domain may in fact be important for the HBV life cycle. For instance, amino acid substitutions in this region may affect the catalytic activity of the polymerase which is essential in the earliest steps in the HBV life cycle. Furthermore, recognition of P antigens may limit early HBV spread and its high degree of conservation may prevent viral escape via mutations in T cell epitopes ([22], [23], see [53] for review). In addition, two studies report the discovery of CD8+ and CD4+ T cell epitopes in the polymerase, although the regions are less than other HBV antigens [23], [24] suggesting that the variation in P may also be associated with limited immune escape.

While it is tempting to associate the observed correlation between positive selection and epitopes with response to host immune pressure as observed in HCV [10], [11], it is important to acknowledge the differences between the HBV and HCV virus life cycles [2] as well as considering the complex interplay between virus and host in HBV necessary for establishing a chronic infection. Multiple factors, including immune evasion, persistence of cccDNA and infection of immunologically privileged sites, contribute to HBV persistence [54]. Chronic infection in HBV is characterized by a weaker immune response [1] and production of excess HBsAg that captures most antibodies and HBV-specific immunosuppression play critical roles in immune evasion [55]. With regards to HBV-specific immunosuppression, from a viral perspective, the various proteins contribute in different ways towards achieving this modulated response. For example, HBeAg can induce tolerance in core protein (HBcAg) specific T cells, reducing their efficacy to kill infected cells [56], [57] and the X protein appears capable of inhibiting antigen processing and presentation, reducing the visibility of infected cells to the immune system [58]. Conversely, from the host perspective, a number of factors have been proposed or demonstrated to be associated with the inadequate cellular immune response including: deficient antigen presentation [59], a limited range of virus-specific T cells [60], anergy or exhaustion of rapid onset of T cell response due to antigen overload and T cell overstimulation [61], induction of regulatory T cells and ramping up of negative regulatory signals such as regulatory T cell mediated immunosuppression [62]. Thus, in a chronic infection, these factors will suppress the host immune response and, consequently, the selection pressure acting on the virus, complicating the interpretation of our results and the significance of our association between positive sites and epitopes.

Interpretation of our results are further confounded by the fact that our dataset represents a broad cross-section of the HBV patient population (comprising chronic infection, acute infection and HBV carriers) and, as such represents an average across multiple patients over the course of an HBV infection. Furthermore, the identified epitope/positive selection signal superimposed over the background noise generated by random mutations due to relaxed selection pressures on flexible loop regions that are free of secondary structure constraints (i.e. α-helix and β-sheet). Finally, it has been proposed that coevolutionary relationship may exist between sites in PreS (corresponding to spacer in P) and sites elsewhere in the genome that are expressed as compensatory mutations. [63].

The variable region (nucleotides 1–498 in the large surface protein reading frame) is essential both for P and S as it encodes the spacer in the P ORF and the PreS region in the S ORF. On one hand, this region provides the flexibility for changes in protein conformation. In P, some residues in TP and RT are believed to be associated with P-ε binding which triggers pregenomic RNA (pgRNA) encapsidation and DNA synthetic priming [64], [65]. This step can proceed only if the P protein changes its conformation from the stable state to the “active” state in the presence of chaperons such as hsp40 and hsp70 [66]–[69]. On the other hand, in S, besides its function in viral entry and infection [70], [71], the peptide from residues 2–48 is the target sequence for the hepatocyte-specific receptor [15]. Also, the flexibility of the Pre-S variable region allows mobilization of this region between the inner and outer face of the virus membrane [72], [73]. This region also has the flexibility to accumulate variation for adapting to immune pressure. PreS harbors many T cell and B cell epitopes for the large surface protein. Hence, adaptive evolution in this region may play a role in HBV escape from the host immune system.

The domain arrangement in P and S further demonstrates the compromise necessary to fulfill the distinct requirements of each protein. The spacer domain in the polymerase (corresponding to PreS in large surface protein) seems to have effectively balanced the distinctive conservation and variation requirements occurring within the overlapping region. Moreover, the four membrane-spanning regions composed of the conserved α-helices contribute to the stability of the S protein, while the intervening variable major hydrophobic regions and C-terminus exposed to the outer face of viral envelope may contribute towards helping HBV evade attack from the host immune system.

In conclusion, our findings indicate the spacer domain, which corresponds to PreS in S, provides an important function by serving as a harbor for maintaining heterogeneity for environmental adaptation as well as providing flexibility for conformational changes and response to immune selective pressure.

## Supporting Information

Table S1 **GenBank accession numbers and genotypes for human HBV sequences.**
(DOC)

Table S2 **S protein epitopes.**
(DOC)

Table S3 **Estimation of PAML parameters for different six sites models of variable ω (** ***dn/ds*** **) among eight HBV genotypes.**
(DOC)

Table S4 **Details of Fisher’s exact test and bivariate logistic regression for results summarized in Table 1.**
(DOC)
