# Supplementary material for: Computational Evolutionary Analysis of the Overlapped Surface (S) and Polymerase (P) Region in Hepatitis B Virus Indicates the Spacer Domain in P Is Crucial for Survival
Source: PLoS One. 2013 Apr 5;8(4):e60098. doi: 10.1371/journal.pone.0060098 (PMC3618453; doi:10.1371/journal.pone.0060098)
Supplement: Table S1 — GenBank accession numbers and genotypes for human HBV sequences. (DOC) [file pone.0060098.s001.doc]

Table S1. GenBank accession numbers and genotypes for human HBV sequences

| Genotype | Accession numbers |
| --- | --- |
| Genotype A  (n=300) | AB014370, AB064314, AB126580, AB194951, AB194952, AB205118, AB222707, AB241114, AB241115, AB300366, AB300367, AB453979, AB453980, AB453981, AB453982,AB453983, AB453984, AB453985, AB453986, AB453987, AB453988, AB453989,AB480036, AB480038, AB480039, AB480040, AB480041, AB549213, AF043560, AF043580, AF090838, AF090839, AF090840, AF090841, AF090842, AF143304, AF462041, AF536524, AF537371, AF537372, AJ309369, AJ309370, AJ344115, AJ627227, AJ627228, AM184125, AM184126, AM282986, AM295795, AM295797, AM295798, AM295799, AM410963, AP007263, AY128092, AY152726, AY161138, AY161139, AY161140, AY161141, AY161145, AY161146, AY233274, AY233275, AY233276, AY233277, AY233278, AY233279, AY233280, AY233281, AY233282, AY233283, AY233284, AY233285, AY233286, AY233287, AY233288, AY233289, AY233290, AY373428, AY373429, AY373432, AY707087, AY738139, AY738140, AY738141, AY738142, AY738143, AY902775, AY903452, AY934763, AY934764, AY934765, AY934766, AY934767, AY934768, AY934769, AY934770, AY934771, AY934772, AY934773, DQ020002, DQ020003, DQ315786, DQ788725, DQ788729, EF208113, EU054331, EU086721, EU366129, EU410082, EU414133, EU594383, EU594384, EU594385, EU594386, EU594387, EU594388, EU594389, EU594390, EU594391, EU594392, EU594393, EU594394, EU594395, EU747320, EU859898, EU859899, EU859900, EU859901, EU859902, EU859903, EU859904, EU859905, EU859906, EU859907, EU859908, EU859909, EU859910, EU859911, EU859912, EU859913, EU859914, EU859915, EU859916, EU859917, EU859918, EU859919, EU859920, EU859921, EU859922, EU859923, EU859924, EU859925, EU859926, EU859927, EU859928, EU859929, EU859930, EU859931, EU859932, EU859933, EU859934, EU859935, EU859936, EU859937, EU859938, EU859939, EU859940, EU859941, EU859942, EU859943, EU859944, EU859945, EU859946, EU859947, EU859948, EU859949, EU859950, EU859951, EU859952, EU859953, EU859954, EU859955, EU859956, FJ349222, FJ349224, FJ349296, FJ692554, FJ692557, FJ692558, FJ692559, FJ692561, FJ692562, FJ692563, FJ692564, FJ692565, FJ692566, FJ692567, FJ692568, FJ692571, FJ692572, FJ692574, FJ692575, FJ692576, FJ692577, FJ692578, FJ692579, FJ692580, FJ692581, FJ692582, FJ692583, FJ692584, FJ692585, FJ692588, FJ692589, FJ692590, FJ692591, FJ692592, FJ692595, FJ692596, FJ692597, FJ692598, FJ692599, FJ692600, FJ692601, FJ692602, FJ692603, FJ692604, FJ692605, FJ692606, FJ692607, FJ692608, FJ692609, FJ692610, FJ692611, FJ692612, FJ692613, FM199974, FM199976, FM199977, FM199978, FM199979, FM199980, FM199981, GQ161813, GQ184323, GQ184324, GQ331046, GQ331047, GQ331048, GQ414522, GQ477460, GQ477461, GQ477462, GQ477463, GQ477464, GQ477465, GQ477466, GQ477467, GQ477468, GQ477469, GQ477471, GQ477472, GQ477473, GQ477475, GQ477477, GQ477478, GQ477479, GQ477480, GQ477481, GQ477482, GQ477483, GQ477484, GQ477485, GQ477486, GQ477487, GQ477488, GQ477489, GQ477490, GQ477491, GQ477492, GQ477493, GQ477494, GQ477495, GQ477496, GQ477497, GQ477498, GQ477500, GQ477501, GQ477502, GQ477503, GQ477504, HM011485, HM363612, HM363613, U87742, X02763, X51970, Z35717 |
| Genotype B  (n=604) | AB010289, AB010290, AB010291, AB010292, AB014366, AB031267, AB033554, AB033555, AB115551, AB117759, AB205119, AB205120, AB205121, AB205122, AB212625, AB219426, AB219427, AB219428, AB219429, AB241117, AB287316, AB287318, AB287319, AB287326, AB287327, AB287328, AB287329, AB300364, AB300371, AB302942, AB302943, AB302944, AB302945, AB540582, AB555498, AB555499, AF121243, AF121244, AF121245, AF121246, AF121247, AF121248, AF121249, AF121250, AF121251, AF282917, AF282918, AF479684, AJ627225, AP011084, AP011085, AP011086, AP011087, AP011088, AP011089, AP011090, AP011091, AP011092, AP011093, AP011094, AP011095, AP011096, AY163869, AY163870, AY167089, AY167093, AY167094, AY167100, AY167101, AY167102, AY206373, AY206375, AY206377, AY206380, AY206383, AY206390, AY206391, AY220697, AY220698, AY220703, AY220704, AY293309, AY518556, AY596102, AY596103, AY596104, AY596105, AY596106, AY596109, AY596110, AY596112, AY766463, AY800389, AY800391, AY800392, D50521, D50522, DQ377158, DQ448619, DQ448620, DQ448621, DQ448622, DQ448623, DQ448624, DQ448625, DQ448626, DQ448627, DQ448628, DQ904357, DQ993680, DQ993681, DQ993683, DQ993684, DQ993685, DQ993686, DQ993697, DQ993698, DQ993699, DQ993700, DQ993701, DQ993702, DQ993703, DQ993704, DQ993705, DQ993706, DQ993707, DQ993708, DQ993709, DQ993710, DQ995801, DQ995802, DQ995804, EF134945, EF134946, EF473971, EF473972, EF473973, EF473974, EF473975, EF473976, EF473977, EF494380, EF494381, EF494382, EU139543, EU158262, EU158263, EU305547, EU305548, EU306678, EU306679, EU306680, EU306681, EU306683, EU306684, EU306695, EU306696, EU306697, EU306699, EU306700, EU306701, EU306702, EU306703, EU306704, EU306705, EU306706, EU306707, EU306708, EU306709, EU306710, EU306711, EU306712, EU330989, EU330990, EU330995, EU330996, EU330997, EU439018, EU439020, EU439022, EU439023, EU439024, EU487256, EU487257, EU522067, EU522072, EU522074, EU547563, EU564822, EU564823, EU564824, EU564826, EU570070, EU570071, EU570075, EU579441, EU589335, EU595030, EU595031, EU660224, EU796066, EU796067, EU796068, EU881997, EU881998, EU882002, EU882003, EU882004, EU919161, EU919162, EU919170, EU919171, EU919172, EU919173, EU919174, EU919175, EU919176, EU939559, EU939633, EU939634, EU939636, EU939638, EU939639, EU939661, EU939663, EU939664, EU939665, EU939666, EU939667, EU939669, EU939670, EU939671, EU939672, EU939673, EU939674, EU939675, EU939676, EU939677, EU939678, FJ032342, FJ032344, FJ032352, FJ032353, FJ032354, FJ032357, FJ032358, FJ349236, FJ386582, FJ386583, FJ386584, FJ386600, FJ386608, FJ386610, FJ386615, FJ386634, FJ386636, FJ386642, FJ386648, FJ386654, FJ386655, FJ386656, FJ386658, FJ386660, FJ386666, FJ386668, FJ386669, FJ386675, FJ386676, FJ386680, FJ386681, FJ386682, FJ386683, FJ386684, FJ386688, FJ518812, FJ562219, FJ562222, FJ562224, FJ562231, FJ562234, FJ562236, FJ562237, FJ562240, FJ562246, FJ562253, FJ562254, FJ562257, FJ562259, FJ562260, FJ562289, FJ562296, FJ562303, FJ562311, FJ562312, FJ562316, FJ562321, FJ562322, FJ787444, FJ787475, FJ787477, FJ899779, FJ899784, FJ899785, FJ899787, FJ899790, FJ899791, GQ205440, GQ475340, GQ924603, GQ924605, GQ924606, GQ924607, GQ924608, GQ924610, GQ924611, GQ924617, GQ924621, GQ924624, GQ924625, GQ924626, GQ924627, GQ924628, GQ924630, GQ924631, GQ924632, GQ924634, GQ924637, GQ924638, GQ924639, GQ924640, GQ924641, GQ924644, GQ924645, GQ924646, GQ924647, GQ924648, GQ924651, GQ924653, GQ924654, GQ924656, GQ924659, GU332690, GU332692, GU332693, GU332695, GU332696, GU332704, GU332705, GU357842, GU434372, GU434373, GU451682, GU815548, GU815549, GU815550, GU815551, GU815552, GU815553, GU815554, GU815555, GU815556, GU815557, GU815558, GU815559, GU815560, GU815561, GU815562, GU815563, GU815564, GU815565, GU815566, GU815567, GU815568, GU815569, GU815570, GU815571, GU815572, GU815574, GU815575, GU815576, GU815577, GU815578, GU815579, GU815581, GU815582, GU815583, GU815584, GU815585, GU815586, GU815587, GU815588, GU815589, GU815590, GU815591, GU815592, GU815593, GU815594, GU815595, GU815596, GU815597, GU815598, GU815599, GU815601, GU815602, GU815603, GU815604, GU815605, GU815606, GU815607, GU815608, GU815609, GU815610, GU815612, GU815613, GU815614, GU815615, GU815617, GU815618, GU815619, GU815620, GU815621, GU815622, GU815623, GU815625, GU815626, GU815627, GU815628, GU815629, GU815630, GU815631, GU815632, GU815633, GU815635, GU815636, GU815637, GU815638, GU815639, GU815640, GU815641, GU815642, GU815643, GU815644, GU815645, GU815647, GU815648, GU815649, GU815650, GU815651, GU815652, GU815653, GU815654, GU815655, GU815656, GU815657, GU815658, GU815659, GU815660, GU815661, GU815662, GU815663, GU815664, GU815665, GU815666, GU815667, GU815668, GU815669, GU815670, GU815672, GU815673, GU815674, GU815675, GU815676, GU815677, GU815678, GU815679, GU815680, GU815681, GU815682, GU815683, GU815684, GU815685, GU815686, GU815687, GU815688, GU815689, GU815690, GU815691, GU815692, GU815693, GU815694, GU815695, GU815696, GU815697, GU815698, GU815699, GU815700, GU815701, GU815702, GU815703, GU815704, GU815705, GU815706, GU815707, GU815708, GU815709, GU815710, GU815711, GU815713, GU815714, GU815715, GU815716, GU815717, GU815718, GU815719, GU815720, GU815721, GU815722, GU815723, GU815724, GU815725, GU815726, GU815727, GU815728, GU815729, GU815730, GU815731, GU815732, GU815733, GU815734, GU815735, GU815736, GU815737, GU815738, GU815739, GU815740, GU815742, GU815743, GU815744, GU815745, GU815746, GU815747, GU815748, GU815749, GU815750, GU815751, GU815752, GU815753, GU815754, GU815755, GU815756, GU815757, GU815758, GU815759, GU815760, GU815761, GU815762, GU815763, GU815764, GU815765, GU815766, GU815767, GU815768, GU815769, GU815770, GU815771, GU815772, GU815773, GU815774, GU815775, GU815776, GU815777, GU815778, GU815779, GU815780, GU815781, GU815782, GU815783, HM011466, HM011467, HM011469, HM011470, HM011471, HM011473, HM011474, HM011475, HM011476, HM011477, HM011480, HM011482, HM011483, HM011484, HM011487, HM011490, HM011492, HM011494, HM011496, HM011498, HM011499, HM011503, HM011504, U87747, X97850, X97851, X98077 |
| Genotype C  (n=717) | AB014360, AB014362, AB014363, AB014364, AB014365, AB014367, AB014369, AB014371, AB014374, AB014376, AB014377, AB014378, AB014379, AB014380, AB014381, AB014382, AB014383, AB014384, AB014385, AB014389, AB014391, AB014392, AB014393, AB014394, AB014396, AB014399, AB031262, AB033550, AB033551, AB033552, AB033553, AB033556, AB033557, AB049609, AB049610, AB074755, AB074756, AB111946, AB112063, AB112065, AB112066, AB112348, AB112408, AB112471, AB112472, AB115417, AB117758, AB205123, AB205124, AB205125, AB205152, AB222714, AB222715, AB247916, AB298720, AB298721, AB299858, AB300359, AB300360, AB300361, AB300362, AB300363, AB300365, AB300368, AB300369, AB300373, AB367392, AB367393, AB367394, AB367395, AB367396, AB367397, AB367398, AB367399, AB367400, AB367401, AB367402, AB367403, AB367404, AB367406, AB367407, AB367410, AB367411, AB367412, AB367414, AB367415, AB367416, AB367417, AB367418, AB367419, AB367420, AB367422, AB367423, AB367424, AB367427, AB367430, AB367431, AB367432, AB367434, AB367435, AB367800, AB367803, AB367804, AB368296, AB368297, AB485808, AB485810, AB540583, AB540584, AB540585, AF068756, AF223954, AF223955, AF223957, AF223958, AF223960, AF223961, AF241410, AF241411, AF286594, AF330110, AF363961, AF384371, AF384372, AF458664, AF458665, AF461357, AF461358, AF461359, AF461361, AF461363, AF473543, AF533983, AP011097, AP011098, AP011099, AP011100, AP011101, AP011102, AP011103, AP011104, AP011105, AP011106, AP011107, AP011108, AY040627, AY057947, AY123041, AY123424, AY167091, AY167095, AY206374, AY206376, AY206379, AY206381, AY206382, AY206384, AY206386, AY206392, AY220699, AY247030, AY247031, AY247032, AY306136, AY596107, AY596108, AY641558, AY641559, AY641560, AY641561, D28880, D50517, D50518, D50519, D50520, DQ089757, DQ089758, DQ089760, DQ089761, DQ089762, DQ089763, DQ089764, DQ089765, DQ089766, DQ089768, DQ089769, DQ089770, DQ089771, DQ089772, DQ089773, DQ089774, DQ089775, DQ089776, DQ089777, DQ089778, DQ089779, DQ089780, DQ089781, DQ089782, DQ089783, DQ089784, DQ089785, DQ089786, DQ089787, DQ089788, DQ089789, DQ089790, DQ089791, DQ089792, DQ089793, DQ089795, DQ089796, DQ089797, DQ089798, DQ089799, DQ089801, DQ089802, DQ089803, DQ089804, DQ246215, DQ315781, DQ315782, DQ315783, DQ377164, DQ377165, DQ478885, DQ478899, DQ683578, DQ890381, DQ922649, DQ922650, DQ922651, DQ975273, DQ980547, DQ993689, DQ993691, DQ993692, DQ993693, EF494379, EF536065, EF536066, EF688062, EU305540, EU305541, EU306673, EU306674, EU306675, EU306685, EU306686, EU306687, EU306688, EU306689, EU306690, EU306691, EU306692, EU306693, EU306694, EU306719, EU306720, EU306721, EU306726, EU306728, EU410079, EU410080, EU439006, EU439015, EU498227, EU522068, EU547558, EU547560, EU547562, EU554535, EU554536, EU554537, EU554538, EU554539, EU554540, EU554541, EU554542, EU560438, EU560439, EU560440, EU560441, EU562215, EU562217, EU562218, EU564820, EU564821, EU570068, EU570072, EU570073, EU570074, EU579442, EU579443, EU589336, EU589337, EU589345, EU660225, EU660226, EU660229, EU670263, EU717212, EU717213, EU787444, EU796070, EU796072, EU871969, EU871970, EU871971, EU871972, EU871973, EU871974, EU871975, EU871976, EU871977, EU871978, EU871979, EU871980, EU871982, EU871984, EU871985, EU871987, EU871988, EU871989, EU871990, EU871992, EU871993, EU871994, EU871996, EU871997, EU871998, EU872000, EU872001, EU872002, EU872003, EU872004, EU872006, EU872017, EU881996, EU882005, EU916210, EU916219, EU916222, EU916223, EU916224, EU916225, EU916232, EU916233, EU916236, EU916237, EU916238, EU916239, EU916240, EU916241, EU919163, EU919168, EU919169, EU939536, EU939538, EU939539, EU939540, EU939542, EU939543, EU939544, EU939545, EU939546, EU939548, EU939550, EU939551, EU939554, EU939556, EU939557, EU939561, EU939562, EU939563, EU939564, EU939566, EU939567, EU939568, EU939570, EU939571, EU939572, EU939578, EU939579, EU939582, EU939585, EU939586, EU939587, EU939588, EU939589, EU939590, EU939591, EU939592, EU939593, EU939594, EU939595, EU939596, EU939597, EU939600, EU939601, EU939603, EU939605, EU939607, EU939609, EU939610, EU939611, EU939612, EU939613, EU939614, EU939615, EU939616, EU939617, EU939618, EU939619, EU939625, EU939626, EU939640, EU939644, EU939647, EU939648, EU939649, EU939651, EU939652, EU939653, EU939654, EU939655, EU939656, EU939657, EU939659, EU939668, FJ032331, FJ032334, FJ032336, FJ032338, FJ032339, FJ032340, FJ032341, FJ032345, FJ032346, FJ032351, FJ032355, FJ032356, FJ032359, FJ032361, FJ349225, FJ386574, FJ386575, FJ386576, FJ386577, FJ386579, FJ386581, FJ386585, FJ386586, FJ386587, FJ386588, FJ386589, FJ386591, FJ386592, FJ386593, FJ386594, FJ386595, FJ386596, FJ386597, FJ386598, FJ386601, FJ386602, FJ386603, FJ386604, FJ386605, FJ386606, FJ386607, FJ386609, FJ386611, FJ386613, FJ386614, FJ386618, FJ386619, FJ386620, FJ386623, FJ386624, FJ386625, FJ386626, FJ386627, FJ386629, FJ386630, FJ386631, FJ386632, FJ386635, FJ386639, FJ386644, FJ386647, FJ386649, FJ386652, FJ386653, FJ386657, FJ386659, FJ386661, FJ386662, FJ386664, FJ386670, FJ386671, FJ386672, FJ386673, FJ386678, FJ386679, FJ386685, FJ386687, FJ386689, FJ518813, FJ562218, FJ562221, FJ562225, FJ562226, FJ562227, FJ562228, FJ562230, FJ562232, FJ562233, FJ562235, FJ562238, FJ562239, FJ562241, FJ562242, FJ562243, FJ562244, FJ562248, FJ562249, FJ562250, FJ562251, FJ562252, FJ562255, FJ562256, FJ562258, FJ562264, FJ562265, FJ562266, FJ562267, FJ562268, FJ562269, FJ562270, FJ562272, FJ562273, FJ562274, FJ562275, FJ562276, FJ562279, FJ562280, FJ562281, FJ562282, FJ562283, FJ562284, FJ562285, FJ562287, FJ562288, FJ562291, FJ562292, FJ562293, FJ562294, FJ562298, FJ562299, FJ562300, FJ562301, FJ562304, FJ562305, FJ562306, FJ562307, FJ562308, FJ562310, FJ562313, FJ562314, FJ562315, FJ562317, FJ562318, FJ562319, FJ562320, FJ562323, FJ562324, FJ562325, FJ562327, FJ562329, FJ562330, FJ562332, FJ562333, FJ562334, FJ562335, FJ562336, FJ562337, FJ787437, FJ787438, FJ787439, FJ787441, FJ787442, FJ787443, FJ787445, FJ787446, FJ787448, FJ787449, FJ787450, FJ787452, FJ787453, FJ787454, FJ787455, FJ787456, FJ787457, FJ787458, FJ787464, FJ787466, FJ787468, FJ787469, FJ787470, FJ787471, FJ787472, FJ787479, FJ787480, FJ787486, FJ899761, FJ899762, FJ899764, FJ899765, FJ899769, FJ899770, FJ899771, FJ899773, FJ899774, FJ899776, FJ899777, FJ899783, FJ899788, FJ899789, GQ184326, GQ205441, GQ259588, GQ372968, GQ475305, GQ475306, GQ475307, GQ475308, GQ475309, GQ475310, GQ475311, GQ475312, GQ475313, GQ475314, GQ475315, GQ475316, GQ475317, GQ475318, GQ475319, GQ475320, GQ475321, GQ475322, GQ475324, GQ475325, GQ475327, GQ475328, GQ475329, GQ475331, GQ475332, GQ475334, GQ475336, GQ475337, GQ475338, GQ475339, GQ475341, GQ475344, GQ475345, GQ475346, GQ475348, GQ475349, GQ475350, GQ475351, GQ475354, GQ475355, GQ475356, GQ475357, GQ872210, GQ872211, GQ924604, GQ924609, GQ924612, GQ924613, GQ924614, GQ924615, GQ924616, GQ924618, GQ924620, GQ924622, GQ924629, GQ924633, GQ924636, GQ924642, GQ924643, GQ924649, GQ924650, GQ924655, GQ924657, GQ924658, GU357843, GU357845, GU385774, GU434374, GU721029, HM011465, HM011468, HM011472, HM011479, HM011481, HM011486, HM011488, HM011489, HM011491, HM011493, HM011495, HM011497, HM011500, HM011501, X52939 |
| Genotype D  (n=323) | AB033558, AB033559, AB104709, AB104710, AB104711, AB104712, AB126581, AB205126, AB205127, AB205128, AB222709, AB222710, AB222711, AB222712, AB222713, AB555496, AB555497, AB555500, AB555501, AF043593, AF043594, AF121239, AF121240, AF121241, AF121242, AF151735, AF280817, AJ131956, AJ344116, AJ344117, AJ627215, AJ627216, AJ627217, AJ627218, AJ627219, AJ627220, AJ627221, AJ627222, AJ627223, AJ627224, AM422939, AY090452, AY090453, AY161150, AY161153, AY161157, AY161158, AY161159, AY161160, AY161161, AY161162, AY161163, AY233291, AY233292, AY233293, AY233294, AY233295, AY233296, AY341335, AY373430, AY741794, AY741795, AY741796, AY741797, AY741798, AY796030, AY796031, AY796032, DQ111986, DQ111987, DQ315776, DQ315778, DQ315779, EU155893, EU414135, EU414136, EU414137, EU414138, EU414139, EU414140, EU414141, EU414142, EU414143, EU594382, EU594396, EU594397, EU594398, EU594399, EU594400, EU594401, EU594402, EU594403, EU594404, EU594405, EU594406, EU594407, EU594408, EU594409, EU594410, EU594415, EU594416, EU594421, EU594422, EU594423, EU594424, EU594425, EU594426, EU594427, EU594428, EU594430, EU594431, EU594432, EU594433, EU594434, EU594435, EU594436, EU787436, EU787437, EU787438, EU787440, EU787441, EU787442, EU787443, EU787446, EU787447, EU919197, EU921418, EU921419, EU939681, FJ349205, FJ349206, FJ349207, FJ349208, FJ349209, FJ349210, FJ349211, FJ349212, FJ349213, FJ349214, FJ349215, FJ349216, FJ349218, FJ349219, FJ349220, FJ349221, FJ349228, FJ349229, FJ349230, FJ349231, FJ349232, FJ349233, FJ349234, FJ349235, FJ386590, FJ562338, FJ899792, FJ904394, FJ904395, FJ904396, FJ904398, FJ904399, FJ904400, FJ904402, FJ904403, FJ904404, FJ904405, FJ904406, FJ904407, FJ904408, FJ904409, FJ904410, FJ904412, FJ904413, FJ904414, FJ904415, FJ904416, FJ904417, FJ904418, FJ904419, FJ904420, FJ904421, FJ904422, FJ904424, FJ904425, FJ904426, FJ904427, FJ904429, FJ904430, FJ904431, FJ904432, FJ904433, FJ904435, FJ904436, FJ904437, FJ904438, FJ904439, FJ904440, FJ904441, FJ904442, FJ904443, FJ904444, FJ904445, FJ904447, GQ167301, GQ167302, GQ183448, GQ183449, GQ183450, GQ183451, GQ183452, GQ183453, GQ183454, GQ183455, GQ183456, GQ183457, GQ183458, GQ183459, GQ183460, GQ183461, GQ183462, GQ183463, GQ183464, GQ183465, GQ183466, GQ183467, GQ183468, GQ183469, GQ183470, GQ183471, GQ183472, GQ183473, GQ183474, GQ183475, GQ183476, GQ183478, GQ183479, GQ183481, GQ183482, GQ183483, GQ183486, GQ184322, GQ205377, GQ205378, GQ205379, GQ205380, GQ205381, GQ205382, GQ205384, GQ205385, GQ205386, GQ205387, GQ205388, GQ205389, GQ477452, GQ477453, GQ477455, GQ477456, GQ477457, GQ477458, GQ477459, GQ922000, GQ922001, GQ922002, GQ922003, GQ922004, GQ922005, GQ924652, GU357846, GU456635, GU456636, GU456637, GU456638, GU456639, GU456640, GU456641, GU456642, GU456643, GU456645, GU456646, GU456647, GU456648, GU456649, GU456650, GU456651, GU456652, GU456653, GU456654, GU456655, GU456656, GU456657, GU456658, GU456659, GU456660, GU456661, GU456662, GU456663, GU456664, GU456665, GU456666, GU456667, GU456668, GU456669, GU456670, GU456673, GU456674, GU456675, GU456676, GU456677, GU456678, GU456679, GU456680, GU456681, GU456682, GU456683, GU456684, X02496, X59795, X65257, X65258, X97848, X97849, Y07587, Z35716 |
| Genotype E  (n=181) | AB032431, AB091255, AB091256, AB106564, AB194947, AB194948, AB205129, AB205188, AB205189, AB205190, AB205191, AB205192, AP007262, AY738144, AY738145, AY738146, AY738147, AY739674, AY739675, AY935700, DQ060822, DQ060823, DQ060824, DQ060825, DQ060826, DQ060827, DQ060828, DQ060829, DQ060830, EU239217, EU239218, EU239219, EU239220, EU239221, EU239222, EU239223, EU239224, EU239225, EU239226, FJ349226, FJ349237, FJ349238, FJ349239, FJ349240, FN594748, FN594749, FN594750, FN594751, FN594752, FN594754, FN594755, FN594756, FN594757, FN594758, FN594759, FN594760, FN594761, FN594762, FN594763, FN594765, FN594766, GQ161755, GQ161757, GQ161758, GQ161759, GQ161760, GQ161761, GQ161762, GQ161763, GQ161764, GQ161765, GQ161766, GQ161768, GQ161769, GQ161770, GQ161771, GQ161772, GQ161773, GQ161774, GQ161775, GQ161776, GQ161777, GQ161778, GQ161779, GQ161780, GQ161781, GQ161782, GQ161783, GQ161784, GQ161785, GQ161786, GQ161787, GQ161789, GQ161790, GQ161791, GQ161792, GQ161793, GQ161794, GQ161796, GQ161797, GQ161798, GQ161799, GQ161800, GQ161801, GQ161802, GQ161803, GQ161804, GQ161805, GQ161807, GQ161808, GQ161809, GQ161810, GQ161811, GQ161812, GQ161814, GQ161815, GQ161816, GQ161817, GQ161818, GQ161819, GQ161820, GQ161821, GQ161824, GQ161825, GQ161826, GQ161827, GQ161828, GQ161829, GQ161830, GQ161831, GQ161832, GQ161833, GQ161834, GQ161835, GQ161836, HM363565, HM363566, HM363567, HM363568, HM363569, HM363570, HM363571, HM363572, HM363573, HM363574, HM363575, HM363576, HM363578, HM363579, HM363580, HM363581, HM363582, HM363583, HM363584, HM363585, HM363586, HM363587, HM363588, HM363589, HM363590, HM363591, HM363592, HM363593, HM363594, HM363595, HM363596, HM363597, HM363598, HM363599, HM363600, HM363601, HM363602, HM363603, HM363604, HM363605, HM363606, HM363607, HM363608, HM363609, HM363610, HM363611 |
| Genotype F  (n=59) | AB036905, AB036906, AB036907, AB036908, AB036909, AB036910, AB036911, AB036912, AB036913, AB036914, AB036915, AB036916, AB036917, AB036918, AB036919, AB036920, AB064316, AB166850, AF223962, AF223963, AF223964, AF223965, AY090455, AY090456, AY090458, AY090459, AY090461, AY179734, AY179735, DQ776247, DQ823086, DQ823087, DQ823088, DQ823089, DQ823090, DQ823091, DQ823092, DQ823093, DQ823094, DQ823095, DQ899142, DQ899143, DQ899144, DQ899145, DQ899146, DQ899147, DQ899148, DQ899149, DQ899150, EU366116, EU366118, EU366132, EU366133, FJ657519, FJ657522, FJ657525, FJ657528, FJ657529, X69798 |
| Genotype G  (n=25) | AB056513, AB056514, AB056515, AB056516, AB064310, AB064311, AB064312, AB064313, AB375165, AB375166, AB375167, AB375168, AB375169, AB375170, AF160501, AF405706, AP007264, DQ207798, EF464097, EF464098, EF464099, EF634480, EF634481, EU833890, GU565217 |
| Genotype H  (n=27) | AB059659, AB059660, AB059661, AB064315, AB179747, AB205010, AB266536, AB298362, AB353764, AB375159, AB375160, AB375161, AB375162, AB375163, AB375164, AB516393, AB516394, AY090454, AY090457, AY090460, EF157291, EU498228, FJ356715, FJ356716, HM066946, HM117850, HM117851 |
