# Supplementary material for: Computational Evolutionary Analysis of the Overlapped Surface (S) and Polymerase (P) Region in Hepatitis B Virus Indicates the Spacer Domain in P Is Crucial for Survival
Source: PLoS One. 2013 Apr 5;8(4):e60098. doi: 10.1371/journal.pone.0060098 (PMC3618453; doi:10.1371/journal.pone.0060098)
Supplement: Table S2 — S protein epitopes. (DOC) [file pone.0060098.s002.doc]

Table S2. epitopes for S protein

| Epitope ID | Linear sequence | sites |
| --- | --- | --- |
| B cell epitopes | | |
| 48335 | PLGFFPDHQLDPAFGANSNNPDWDFN | 10-35 |
| 51307 | QLDPAFGA | 18-25 |
| 9586 | DPAFR | 20-24 |
| 4802 | ASTNRQSGRQPTPISPPLRDSHPQ | 84-107 |
| 42443 | MQWNSTTFHQTLQDPRVRGLYFPAGG | 109-134 |
| 46013 | NSTTFHQTLQDPRVRGLYFPAGGSSSGTVNPVPTTVSPISSIFSRIGD | 112-159 |
| 6529 | CKTCTTPAQGNSMFPSCCCTKPTDGNCTCIPIPSSWAFAKYLWEWAS | 284-330 |
| 63086 | TCMTTAQGTSMYPSC | 286-300 |
| 7161 | CTIPAQGTSMFPSCCCTKPSDGNC | 287-310 |
| 7171 | CTKPTDGNC | 302-310 |
| 47591 | PFVQWFMGL | 341-349 |
| HLAI T cell epitopes | | |
| 42430 | MQWNSTAFHQTLQDP | 109-123 |
| 37170 | LLDPRVRGL | 120-128 |
| 2002 | AILSKTGDPV | 152-161 |
| 69714 | VLQAGFFIL | 177-185 |
| 16753 | FLLTKILTI | 183-191 |
| 62504 | SWWTSLNFL | 197-205 |
| 16617 | FLGGTRVCL | 204-212 |
| 27168 | ILLLCLIFL | 249-257 |
| 27169 | ILLLCLIFLL | 249-258 |
| 37466 | LLLCLIFLL | 250-258 |
| 37120 | LLCLIFLLV | 251-259 |
| 79601 | LLCLIFLLVL | 251-260 |
| 40447 | LVLLDYQGML | 258-267 |
| 69581 | VLLDYQGML | 259-267 |
| 37187 | LLDYQGMLPV | 260-269 |
| 27878 | IPIPSSWAF | 313-321 |
| 62477 | SWLSLLVPF | 334-342 |
| 72794 | WLSLLVPFV | 335-343 |
| 37919 | LLVPFVQWFV | 338-347 |
| 21139 | GLSPTVWLSV | 348-357 |
| 58740 | SIVSPFIPLL | 370-379 |
| 27345 | ILSPFLPLL | 371-379 |
| HLAII T cell epitopes | | |
| 48330 | PLGFFPDHQL | 41201 |
| 48333 | PLGFFPDHQLDPAFGA | 41207 |
| 51310 | QLDPAFRANTANPDWDFNPN | 18-37 |
| 3966 | AQGILQTLPANPPPA | 70-84 |
| 46959 | PASTNRQSGRRQPTPL | 83-98 |
| 42441 | MQWNSTTFHQTLQ | 109-121 |
| 42430 | MQWNSTAFHQTLQDP | 109-123 |
| 61342 | SSSGTVNPAPNIASHISSIS | 135-154 |
| 58034 | SGFLGPLLVLQAGFFLLTR | 169-187 |
| 50262 | QAGFFLLTRILTIPQS | 179-194 |
| 50259 | QAGFFLLTKILTIPLSLD | 179-196 |
| 1505 | AGFFLLTRILTIPQS | 180-194 |
| 15848 | FFLLTRILTI | 182-191 |
| 15849 | FFLLTRILTIPQSLD | 182-196 |
| 37870 | LLTRILTI | 184-191 |
| 66038 | TRILTIPQSLDSWWTSLNF | 186-204 |
| 10274 | DSWWTSLNFLGGSPVCLGQN | 196-215 |
| 66307 | TSLNFLGGSPVC | 200-211 |
| 59353 | SLNFLGGTTVCLGQN | 201-215 |
| 6574 | CLGQNSQSPTSNHSPTSCPPTCPGYRWMCLRRFI | 211-244 |
| 47877 | PICPGYRWMCLRRFIIFL | 230-247 |
| 26485 | IIFLFILLLCLIFLLVLLD | 244-262 |
| 21305 | GMLPVCPLIPGSTTTNTG | 265-282 |
| 66746 | TTNTGPCKTCTTPAQG | 278-293 |
| 7230 | CTTPAQGNSMFPSC | 287-300 |
| 7233 | CTTPAQGNSMFPSCCCTKPTDGNC | 287-310 |
| 57067 | SCCCTKPTDGNCTCIPIPSS | 299-318 |
| 7171 | CTKPTDGNC | 302-310 |
| 15209 | FAKYLWEWASVRFSWLSL | 321-338 |
| 72385 | WEWASVRFSWLS | 326-337 |
| 72263 | WASVRFSW | 328-335 |
| 72265 | WASVRFSWLSLLVPFV | 328-343 |
| 62310 | SVRFSWLSLLVPFVQWF | 330-346 |
| 62479 | SWLSLLVPFVQWFVGL | 334-349 |
| 70295 | VPFVQWFVGLSPTVWLSA | 340-357 |
| 76458 | YWGPSLYSIVSPFIPL | 363-378 |
| 17331 | FPLLPIFFCLWVYI | 376-389 |
| 37664 | LLPIFFCLW | 378-386 |
