# Supplementary material for: Computational Evolutionary Analysis of the Overlapped Surface (S) and Polymerase (P) Region in Hepatitis B Virus Indicates the Spacer Domain in P Is Crucial for Survival
Source: PLoS One. 2013 Apr 5;8(4):e60098. doi: 10.1371/journal.pone.0060098 (PMC3618453; doi:10.1371/journal.pone.0060098)
Supplement: Table S3 — Estimation of PAML parameters for different six sites models of variable ω ( dn/ds ) among eight HBV genotypes. (DOC) [file pone.0060098.s003.doc]

Table S3. PAML parameters estimation for six sites models of variable ω (*dn/ds*) among eight HBV genotypes

| Gene | M0 | M1 | M2 | M3 | M7 | M8 | M3 vs. M0  df=4  p (χ^2^ test) | M2 vs. M1  df=2  p (χ^2^ test) | M8 vs. M7  df=2  p (χ^2^ test) |
| --- | --- | --- | --- | --- | --- | --- | --- | --- | --- |
| Genotype A parameter estimates | | | | | | | | | |
| S | lnL=-5260.76  ω= 0.686 | lnL=-5194.91  p_0_=0.564,ω_0_=0.061  p_1_=0.436,ω_1_=1 | lnL=-5159.21  p_0_=0.552,ω_0_=0.086  p_1_=0.394,ω_1_=1  p_2_=0.054,ω_2_=4.687 | lnL=-5158.17  p_0_=0.716,ω_0_=0.179  p_1_=0.256,ω_1_=1.624  p_2_=0.027,ω_2_=6.123 | lnL=-5195.78  p=0.017  q=0.015 | lnL=-5159.53  p=0.142  q=0.165  ω= 4.592 | 2.89E-43** | 3.13E-16** | 1.81E-16** |
| P | lnL=-11613.89  ω= 0.325 | lnL=-11434.44  p_0_=0.798,ω_0_=0.098  p_1_=0.202,ω_1_=1 | lnL=-11413.65  p_0_=0.808,ω_0_=0.114  p_1_=0.166,ω_1_=1  p_2_=0.026,ω_2_=2.995 | lnL=-11413.37  p_0_=0.775,ω_0_=0.097  p_1_=0.205,ω_1_=0.762  p_2_=0.039,ω_2_=2.659 | lnL=-11448.11  p=0.205  q=0.506 | lnL=-11413.95  p=0.467  q=1.610  ω= 2.460 | 1.66E-85** | 9.35E-10** | 1.46E-15** |
| Genotype B parameter estimates | | | | | | | | | |
| S | lnL=-5846.76  ω= 0.623 | lnL=-5737.42  p_0_=0.642,ω_0_=0.065  p_1_=0.358,ω_1_=1 | lnL=-5672.67  p_0_=0.594,ω_0_=0.064  p_1_=0.363,ω_1_=1  p_2_=0.043,ω_2_=5.884 | lnL=-5672.61  p_0_=0.620,ω_0_=0.078  p_1_=0.339,ω_1_=1.071  p_2_=0.041,ω_2_=5.998 | lnL=-5739.56  p=0.020  q=0.025 | lnL=-5672.95  p=0.112  q=0.158  ω= 5.796 | 4.08E-74** | 7.58E-29** | 1.18E-29** |
| P | lnL=-12964.89  ω= 0.358 | lnL=-12630.80  p_0_=0.756,ω_0_=0.055  p_1_=0.244,ω_1_=1 | lnL=-12569.76  p_0_=0.743,ω_0_=0.114  p_1_=0.233,ω_1_=1  p_2_=0.024,ω_2_=4.134 | lnL=-12569.76  p_0_=0.742,ω_0_=0.059  p_1_=0.234,ω_1_=0.993  p_2_=0.024,ω_2_=4.122 | lnL=-12642.52  p=0.105  q=0.260 | lnL=-12572.76  p=0.143  q=0.389  ω= 3.866 | 9.89E-170** | 3.09E-27** | 5.05E-31** |
| Genotype C parameter estimates | | | | | | | | | |
| S | lnL=-8949.66  ω= 0.593 | lnL=-8773.91  p_0_=0.724,ω_0_=0.146  p_1_=0.276,ω_1_=1 | lnL=-8666.78  p_0_=0.694,ω_0_=0.179  p_1_=0.254,ω_1_=1  p_2_=0.052,ω_2_=4.762 | lnL=-8666.67  p_0_=0.663,ω_0_=0.165  p_1_=0.283,ω_1_=0.915  p_2_=0.054,ω_2_=4.651 | lnL=-8792.48  p=0.303  q=0.443 | lnL=-8667.87  p=0.595  q=0.991  ω= 4.439 | 3.57E-121** | 2.98E-47** | 7.63E-55** |
| P | lnL=-18921.98  ω= 0.362 | lnL=-18323.35  p_0_=0.779,ω_0_=0.076  p_1_=0.221,ω_1_=1 | lnL=-18226.14  p_0_=0.768,ω_0_=0.114  p_1_=0.193,ω_1_=1  p_2_=0.039,ω_2_=3.303 | lnL=-18225.89  p_0_=0.759,ω_0_=0.081  p_1_=0.199,ω_1_=0.935  p_2_=0.042,ω_2_=3.185 | lnL=-18360.41  p=0.183  q=0.479 | lnL=-18234.93  p=0.293  q=0.998  ω= 2.726 | 3.43E-300** | 6.06E-43** | 3.19E-55** |
| Genotype D parameter estimates | | | | | | | | | |
| S | lnL=-5951.69  ω= 0.454 | lnL=-5851.61  p_0_=0.729,ω_0_=0.092  p_1_=0.271,ω_1_=1 | lnL=-5829.71  p_0_=0.753,ω_0_=0.121  p_1_=0.189,ω_1_=1  p_2_=0.059,ω_2_=3.020 | lnL=-5830.86  p_0_=0.232,ω_0_=0  p_1_=0.633,ω_1_=0.256  p_2_=0.135,ω_2_=2.270 | lnL=-5859.42  p=0.143  q=0.255 | lnL=-5830.84  p=1.015  q=3.965  ω= 2.338 | 4.07E-51** | 3.08E-10** | 3.87E-13** |
| P | lnL=-13846.02  ω= 0.358 | lnL=-13483.34  p_0_=0.781,ω_0_=0.069  p_1_=0.219,ω_1_=1 | lnL=-13433.85  p_0_=0.779,ω_0_=0.079  p_1_=0.182,ω_1_=1  p_2_=0.038,ω_2_=3.072 | lnL=-13426.81  p_0_=0.825,ω_0_=0.099  p_1_=0.169,ω_1_=1.515  p_2_=0.006,ω_2_=6.276 | lnL=-13505.79  p=0.136  q=0.349 | lnL=-13439.10  p=0.285  q=1.108  ω= 2.481 | 3.65E-180** | 3.21E-22** | 1.09E-29** |
| Genotype E parameter estimates | | | | | | | | | |
| S | lnL=-2973.71  ω= 0.745 | lnL=-2959.92  p_0_=0.486,ω_0_=0  p_1_=0.514,ω_1_=1 | lnL=-2951.05  p_0_=0.704,ω_0_=0.160  p_1_=0.147,ω_1_=1  p_2_=0.149,ω_2_=3.520 | lnL=-2951.04  p_0_=0.397,ω_0_=0  p_1_=0.455,ω_1_=0.565  p_2_=0.148,ω_2_=3.550 | lnL=-2959.54  p=0.005  q=0.005 | lnL=-2951.05  p=0.442  q=1.028  ω= 3.526 | 3.38E-9** | 0.0001** | 0.0002** |
| P | lnL=-7508.37  ω= 0.323 | lnL=-7422.69  p_0_=0.742,ω_0_=0.029  p_1_=0.258,ω_1_=1 | lnL=-7406.98  p_0_=0.746,ω_0_=0.039  p_1_=0.242,ω_1_=1  p_2_=0.012,ω_2_=5.413 | lnL=-7406.92  p_0_=0.691,ω_0_=0.014  p_1_=0.294,ω_1_=0.850  p_2_=0.015,ω_2_=5.013 | lnL=-7423.35  p=0.012  q=0.024 | lnL=-7406.91  p=0.071  q=0.194  ω= 5.150 | 8.94E-43** | 1.50E-7** | 7.25E-8** |
| Genotype F parameter estimates | | | | | | | | | |
| S | lnL=-2974.32  ω= 0.258 | lnL=-2959.36  p_0_=0.777,ω_0_=0.034  p_1_=0.223,ω_1_=1 | lnL=-2957.94  p_0_=0.809,ω_0_=0.060  p_1_=0.182,ω_1_=1  p_2_=0.009,ω_2_=4.655 | lnL=-2957.93  p_0_=0.650,ω_0_=0  p_1_=0.333,ω_1_=0.869  p_2_=0.017,ω_2_=3.690 | lnL=-2959.53  p=0.026  q=0.078 | lnL=-2957.93  p=0.130  q=0.455  ω= 4.089 | 1.33E-6** | 0.243^NS^ | 0.203^NS^ |
| P | lnL=-7345.33  ω= 0.320 | lnL=-7258.71  p_0_=0.741,ω_0_=0.025  p_1_=0.259,ω_1_=1 | lnL=-7248.23  p_0_=0.784,ω_0_=0.057  p_1_=0.184,ω_1_=1  p_2_=0.032,ω_2_=3.371 | lnL=-7248.19  p_0_=0.740,ω_0_=0.041  p_1_=0.221,ω_1_=0.817  p_2_=0.039,ω_2_=3.177 | lnL=-7259.32  p=0.009  q=0.019 | lnL=-7248.21  p=0.139  q=0.501  ω= 3.131 | 6.38E-41** | 2.81E-5** | 1.50E-5** |
| Genotype G parameter estimates | | | | | | | | | |
| S | lnL=-2334.77  ω= 0.619 | lnL=-2329.73  p_0_=0.486,ω_0_=0  p_1_=0.514,ω_1_=1 | lnL=-2321.26  p_0_=0.443,ω_0_=0.086  p_1_=0.554,ω_1_=1  p_2_=0.003,ω_2_=49.99 | lnL=-2320.66  p_0_=0.595,ω_0_=0.179  p_1_=0.403,ω_1_=1.558  p_2_=0.002,ω_2_=66.02 | lnL=-2329.75  p=0.008  q=0.008 | lnL=-2321.44  p=0.005  q=0.005  ω= 47.64 | 1.13E-05** | 0.0002** | 0.0002** |
| P | lnL=-4791.21  ω= 0.577 | lnL=-4776.38  p_0_=0.568,ω_0_=0  p_1_=0.432,ω_1_=1 | lnL=-4765.31  p_0_=0.555,ω_0_=0  p_1_=0.421,ω_1_=1  p_2_=0.024,ω_2_=9.21 | lnL=-4765.28  p_0_=0.604,ω_0_=0.097  p_1_=0.377,ω_1_=1.204  p_2_=0.019,ω_2_=10.34 | lnL=-4776.56  p=0.005  q=0.007 | lnL=-4765.34  p=0.015  q=0.019  ω= 8.871 | 1.48E-10** | 1.56E-5** | 1.34E-5** |
| Genotype H parameter estimates | | | | | | | | | |
| S | lnL=-2106.99  ω= 0.565 | lnL=-2103.44  p_0_=0.544,ω_0_=0  p_1_=0.456,ω_1_=1 | lnL=-2100.79  p_0_=0.951,ω_0_=0.316  p_1_=0,ω_1_=1  p_2_=0.049,ω_2_=5.722 | lnL=-2100.79  p_0_=0.529,ω_0_=0.316  p_1_=0.421,ω_1_=0.316  p_2_=0.049,ω_2_=5.722 | lnL=-2103.52  p=0.005  q=0.005 | lnL=-2100.79  p=45.76  q=98.89  ω=5.731 | 0.015* | 0.071^NS^ | 0.065^NS^ |
| P | lnL=-4975.57  ω= 0.303 | lnL=-4950.38  p_0_=0.744,ω_0_=0  p_1_=0.256,ω_1_=1 | lnL=-4945.25  p_0_=0.937,ω_0_=0.122  p_1_=0,ω_1_=1  p_2_=0.063,ω_2_=3.296 | lnL=-4945.25  p_0_=0.192,ω_0_=0.122  p_1_=0.745,ω_1_=0.122  p_2_=0.063,ω_2_=3.296 | lnL=-4951.18  p=0.005  q=0.012 | lnL=-4945.26  p=13.902  q=99.00  ω= 3.301 | 2.13 E-12** | 0.006** | 0.003** |

NSsites model (0:one w; 1:neutral; 2:selection; 3:discrete; 7:beta; 8:beta&w). NS: not significant
