# Supplementary material for: Computational Evolutionary Analysis of the Overlapped Surface (S) and Polymerase (P) Region in Hepatitis B Virus Indicates the Spacer Domain in P Is Crucial for Survival
Source: PLoS One. 2013 Apr 5;8(4):e60098. doi: 10.1371/journal.pone.0060098 (PMC3618453; doi:10.1371/journal.pone.0060098)
Supplement: Table S4 — Details of Fisher’s exact test and bivariate logistic regression for results summarized in Table 1. (DOC) [file pone.0060098.s004.doc]

**Table S4**

**Contingency tables for Fisher’s exact test in Table 1.**

Conservation VS 2^nd^ structure in P protein

|  | Conserved | Non-conserved |
| --- | --- | --- |
| α-helix | 102 | 28 |
| β-sheet | 25 | 10 |
| Non-structural site | 126 | 98 |

Conservation VS 2^nd^ structure in S protein

|  | Conserved | Non-conserved |
| --- | --- | --- |
| α-helix | 98 | 22 |
| β-sheet | 12 | 5 |
| Non-structural site | 175 | 77 |

Positive selection sites VS epitope in S

|  | epitope | Non-epitope |
| --- | --- | --- |
| Positive selection site | 31 | 0 |
| Non-positive selection site | 304 | 54 |

Variation VS epitope in S

|  | Variable site | Non-variable site |
| --- | --- | --- |
| epitope | 82 | 253 |
| Non-epitope | 22 | 32 |

**Logistic Regression information for Table 2**

Table 2A . Logistic regression analysis was performed with:

predicted variable Y = conserved sites, X1 = α-helix and β-sheet, X2 = epitope

Table 2B. Logistic regression analysis was performed with

predicted variable Y = positive selection sites, X1 = α-helix and β-sheet, X2 = epitope

with data in the format

| site position | y | x1 | x2 |
| --- | --- | --- | --- |
| 1 | 0 | 0 | 0 |
| 2 | 0 | 0 | 0 |
| 3 | 0 | 0 | 0 |
| 4 | 1 | 0 | 0 |
| … |  |  |  |

where x1=1 indicates the site is located within an α-helix or β-sheet, and x2=1 indicates the site is located within an epitope.
